# *Helicobacter pylori* Colonization Drives Urokinase Receptor (uPAR) Expression in Murine Gastric Epithelium During Early Pathogenesis

**DOI:** 10.3390/microorganisms8071019

**Published:** 2020-07-09

**Authors:** Warner Alpízar-Alpízar, Mette E. Skindersoe, Lone Rasmussen, Mette C. Kriegbaum, Ib J. Christensen, Ida K. Lund, Martin Illemann, Ole D. Laerum, Karen A. Krogfelt, Leif P. Andersen, Michael Ploug

**Affiliations:** 1 The Finsen Laboratory, Rigshospitalet, 2100 Copenhagen, Denmark; mette.kriegbaum@hotmail.com (M.C.K.); ib.jarle.christensen@regionh.dk (I.J.C); ik_lund@hotmail.com (I.K.L.); martinillemann@yahoo.com (M.I.); ole.larum@uib.no (O.D.L.); 2 Biotech Research and Innovation Centre (BRIC), University of Copenhagen, 2100 Copenhagen, Denmark; 3 Centre for Research on Microscopic Structures (CIEMic) and Department of Biochemistry, University of Costa Rica, 2060 San José, Costa Rica; 4 Department of Bacteria, Parasites and Fungi, Statens Serum Institute, 2300 Copenhagen, Denmark; mskindersoe@gmail.com (M.E.S.); kak@ssi.dk (K.A.K.); 5 Bacthera, Kogle Allé 6, 2970 Hoersholm, Denmark; 6 Department of Clinical Microbiology, Rigshospitalet, 2100 Copenhagen, Denmark; leifpercivalandersen@outlook.dk (L.P.A.); lone@jth.dk (L.R.); 7 Hvidovre Hospital, University of Copenhagen, 2650 Copenhagen, Denmark; 8 Department of Science and Environment, Roskilde University, 4000 Roskilde, Denmark; 9 Department of Virus and microbiological Diagnostics, Statens Serum Institute, 2300 Copenhagen, Denmark

**Keywords:** uPAR, *Helicobacter pylori*, gastric cancer, gastritis, mucous metaplasia, mouse model

## Abstract

(1) Background: Persistent *Helicobacter pylori* infection is the most important risk factor for gastric cancer. The urokinase receptor (uPAR) is upregulated in lesions harboring cancer invasion and inflammation. Circumstantial evidence tends to correlate *H. pylori* colonization with increased uPAR expression in the human gastric epithelium, but a direct causative link has not yet been established in vivo; (2) Methods: In a mouse model of *H. pylori*-induced gastritis, we investigated the temporal emergence of uPAR protein expression in the gastric mucosa in response to *H. pylori* (SS1 strain) infection; (3) Results: We observed intense uPAR immunoreactivity in foveolar epithelial cells of the gastric corpus due to de novo synthesis, compared to non-infected animals. This uPAR induction represents a very early response, but it increases progressively over time as do infiltrating immune cells. Eradication of *H. pylori* infection by antimicrobial therapy causes a regression of uPAR expression to its physiological baseline levels. Suppression of the inflammatory response by prostaglandin E_2_ treatment attenuates uPAR expression. Notwithstanding this relationship, *H. pylori* does induce uPAR expression in vitro in co-cultures with gastric cancer cell lines; (4) Conclusions: We showed that persistent *H. pylori* colonization is a necessary event for the emergence of a relatively high uPAR protein expression in murine gastric epithelial cells.

## 1. Introduction

Gastric cancer is the final clinical endpoint of a stepwise process in which *Helicobacter pylori* infection plays a central role [1,2]. Other gastric pathologies associated with *H. pylori* infection include peptic ulcer disease and mucosa-associated lymphoid tissue (MALT) lymphomas [3]. This bacterial infection has also been inversely associated with a range of extra-gastric systemic manifestations, including asthma and allergies [4,5]. The infection is usually established early in life and persists lifelong in the absence of treatment. This leads to a sustained chronic inflammation characterized by infiltration of inflammatory cells in the gastric mucosa and expression of inflammatory mediators by immune and epithelial cells [6,7]. Certain *H. pylori* strains are associated with a higher risk of gastric cancer, in particular those that carry specific virulence factors, such as the vacuolating cytotoxin (VacA) and cytotoxin-associated gene A (CagA) [8]. The combination of bacterial factors, host immune response, and environmental insults drives the initiation and progression from mucosal atrophy, intestinal metaplasia, and dysplasia towards gastric cancer—a stepwise process known as the Correa cascade [1,9]. How *H. pylori* and its virulence factors interfere with the physiological processes as well as the biological mechanisms determining the final outcome remain largely unknown.

Extravascular activation of plasminogen is controlled by the urokinase-type plasminogen activator (uPA), its receptor (uPAR), its inhibitor PAI-1, and α_2_-antiplasmin [10]. Besides degrading major extracellular matrix proteins (e.g., fibrin), the generated plasmin also releases latent growth factors sequestered in the matrix [8]. Several studies correlate uPAR expression in cancer lesions with invasive and metastatic disease, and levels of shed soluble uPAR in plasma correlate accordingly with cancer patient survival in various types of cancer, including gastric cancer [11,12,13]. Mechanistic studies have shown that overexpression of uPAR in tumor cells confers enhanced proliferative, inva-sive, and metastatic potential through the interaction of uPAR with integrins and fibronectin [14,15,16,17,18]. Large research efforts are currently being devoted to the development of a non-invasive imaging platform for uPAR expression by positron emission tomography with a high clinical translational potential [19,20,21]. Recently, uPAR emerged as a cell-surface protein induced during senescence and a potential target for chimeric antigen receptor (CAR) T cell-based therapy for senescence-associated diseases [22].

Accumulating experimental evidence tends to implicate *H. pylori* in the induction of uPAR expression both in vitro and in vivo. Global gene-profiling studies highlight uPAR as one of the top upregulated genes in AGS and T84 cell lines, when co-cultured with *H. pylori* [23,24,25,26]. Clinical studies show increased global uPAR mRNA levels in biopsies of the gastric corpus of *H. pylori*-infected patients [27]. In general, studies on global mRNA expression levels nonetheless often lack information on histological confinement of the expression and furthermore have the inherent caveat that mRNA levels rarely correlate with actual protein levels [28]. We reported a correlation between uPAR protein expression in foveolar epithelial cells and the presence of *H. pylori* in human gastric mucosa [29]. Nevertheless, de novo synthesis of uPAR protein in the gastric epithelium in response to a *H. pylori* challenge has, to the best of our knowledge, never been systematically studied in vivo. The present study aimed to establish the in vivo kinetics of uPAR induction in the gastric mucosa in response to *H. pylori* infection and to explore the correlation to inflammation and gastric pathology in a mouse model of *H. pylori*-induced gastritis. We now demonstrate that sustained *H. pylori* colonization of the gastric mucosa is a necessary event for the induction of relatively high uPAR protein expression. The functional implications of our finding need to be further investigated given the central role of uPAR in cancer biology, and the circumstantial evidence from mouse models of pulmonary infections implicating uPAR in the host clearance of the causative bacterial pathogens [30,31,32,33].

## 2. Material and Methods

### 2.1. Ethics Statement

All murine experiments included in this study were carried out in accordance with the principles of the Basel Declaration and recommendations of the Danish Animal Experiments Inspectorate and the Department of Experimental Medicine (AEM). The protocol was approved by the Danish Animal Experiments Inspectorate and the AEM (permission number: 2010/561-1394 and project number: P12-165, respectively).

### 2.2. Mice

Five- to six-week-old female pathogen-free C57BL/6 mice were obtained from Taconic (Silkeborg, Denmark) and housed under specific pathogen-free conditions (including *H. pylori* and other *Helicobacter* species) at the Animal Facility of the AEM, University of Copenhagen. Mice were offered sterile autoclaved food (Laboratory Autoclavable Rodent Diet 5010, Code: 0001326, LabDiet) and water ad libitum. Mice enrolled in the experiments were between 6 and 8 weeks old and were moved to an experimental section before they were inoculated with *H. pylori*. Of note, the animal facility has a thorough monitoring program to check for pathogens.

### 2.3. H. pylori Culture and Experimental Infections

**Figure 1:**
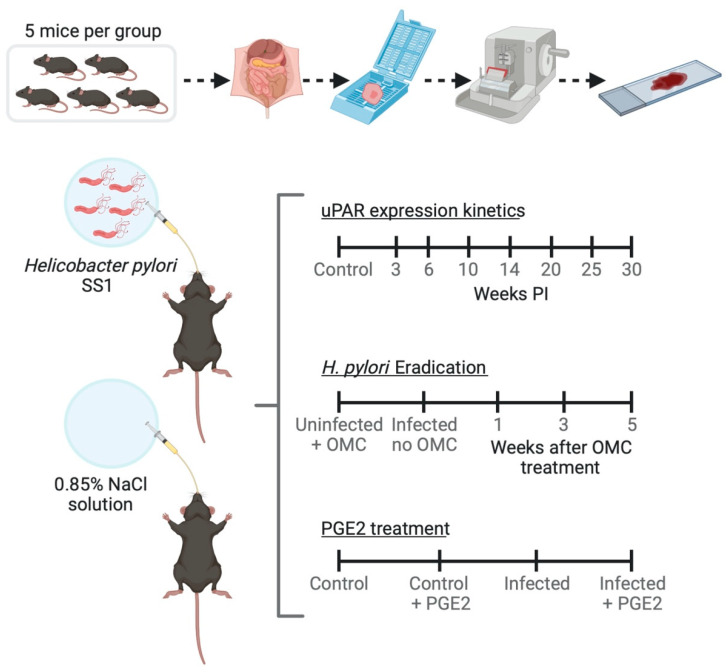
Schematic view of the experiments performed on a mouse model of *Helicobacter pylori*-induced gastritis. OMC; omeprazole, metronidazole, clarithromycin; PGE2; Prostaglandin E_2_ (created with BioRender.com).

Two *H. pylori* strains, SS1 (mouse-adapted *cagA*+, *vacA*+ strain with no functionality of the *cag* pathogenicity island (*cag* PAI)) and J99 (*cagA*+, *vacA*+), were grown on chocolate agar plates at 37 °C under microaerobic conditions (5% O_2_, 10% CO_2_, and 85% N_2_). Forty-eight hours after the first sub-cultivation, the SS1 strain was harvested and suspended in 0.85% (*w/v*) NaCl Medium (bioMérieux, Marcy l’Etoile, France) and adjusted to a McFarland turbidity standard of 3 (≈10^8^ CFU/mL). Mice were inoculated with a single dose of approximately 10^8^ bacteria (CFU) in 0.2 mL of 0.85% NaCl by oral gavage (20G, 3.8-cm disposable animal feeding needles, Code: 01-208-87, Fisher Scientific, Waltham, MA, United States). Control mice received 0.2 mL of saline (Figure 1).

### 2.4. Antimicrobial Therapy for H. pylori Eradication in Mice

Fourteen weeks post-inoculation (PI), animals (*n* = 5 mice per experimental group) received antimicrobial therapy against *H. pylori* consisting of Omeprazole (400 µmol/kg/day; Sigma Aldrich, Søborg, Denmark), Metronidazole (14.2 mg/kg/day; Baxter, Søborg, Denmark), and Clarithromycin (7.15 mg/kg/day; Sigma Aldrich, Søborg, Denmark) once a day for 7 days (Figure 1). A similar antimicrobial therapy has been used successfully to eradicate *H. pylori* from experimentally infected mice [34,35]. Stocks and working dilutions were prepared as described [35].

### 2.5. Pharmacological Treatment of Mice with Prostaglandin E_2_ (PGE_2_) Analogs

Five day PI animals (*n* = 5 mice per experimental group) were treated with a mixture of 16,16-dimethyl PGE_2_ and 17-phenyl trinor PGE_2_ (Cayman Chemical, Ann Arbor, MI, USA) or with phosphate-buffered saline (PBS) for 14 weeks by dosing twice intraperitoneally (i.p.) and once combined i.p./orally per week (Figure 1) as described [36]. Doses of 30 µg/week/mouse of the mixture of PGE_2_ analogs were administered [36].

### 2.6. Resection and Processing of Gastric Tissue for Histology

For the isolation of stomach tissue, mice were initially anaesthetized by i.p. injection of 0.1 mL/10 g of a 1:1 mixture of Dormicum (Midazolam 5 mg/mL) and Hypnorm (Fluanison 5 mg/mL and Fentanyl 0.1 mg/mL), and subsequently perfused with cold PBS, followed by perfusion with 4% paraformaldehyde in PBS. Resected stomachs were opened along the greater curvature, washed with PBS, and cut longitudinally into four stripes, extending from the squamous forestomach through the duodenum. These stripes were fixed overnight in 4% paraformaldehyde and paraffin embedded. Then, 3-µm tissue sections were deparaffinized in xylene and hydrated in a gradual series of ethanol-water dilutions. Sections were stained either with hematoxylin and eosin (H&E) or Alcian Blue and Periodic Acid Schiff (AB/PAS).

### 2.7. Immunohistochemical Detection of uPAR, Ki67, and Inflammation Markers in Gastric Mucosa

Immunohistochemical stainings were performed with the following antibodies: Rabbit polyclonal (pAb) against mouse uPAR [37]; rabbit pAb against *H. pylori* (Code: B0471), rat anti-mouse Ki67 monoclonal antibody (mAb) (Code: M7249), and pAb against human CD3 (Code: A0452) (Dako, Glostrup, Denmark); and rat anti-mouse F4/80 mAb (Code: Ab6640, Abcam, Cambridge, UK). Sections were pre-treated with Proteinase K (10 μg/mL) for 10 and 5 min at 37 °C for antigen retrieval of uPAR and F4/80, respectively. For *H. pylori* immunohistochemistry, sections were treated in a T/T Micromed microwave processor (Milestone, Sorisol, Italy) at 98 °C for 10 min in target retrieval solution pH 6.0 (Code: 1699, Dako, Glostrup, Denmark). For Ki67 and CD3 staining, tissue sections were treated at 98 °C for 15 min in 10 mM sodium citrate pH 6.0 and 10 min in 10 mM Tris, 0.5 mM EGTA (TEG) at pH 9.0, respectively. In all cases, endogenous peroxidase activity was blocked by incubation in 1% hydrogen peroxide solution for 15 min. The primary antibodies were diluted in Antibody Diluent (Code: S3022, Dako, Glostrup, Denmark) and incubated at 4 °C overnight in Shandon racks (Thermo Shandon, Pittsburg, PA, USA) at the following dilutions: Anti-mouse uPAR 1 µg/mL, anti-*H. pylori* 1:700, anti-mouse Ki67 1:200, anti-human CD3 1:2000, and anti-mouse F4/80 1:7000. Subsequently, the primary antibodies against mouse uPAR, *H. pylori*, and CD3 were detected with EnVision reagent anti-rabbit IgG horseradish peroxidase-conjugated polymers (Code: K4003, Dako, Glostrup, Denmark). The rat anti-mouse Ki67 and F4/80 primary antibodies were detected by incubating the sections for 45 min with rabbit anti-rat immunoglobulins/Biotinylated (Code: E0468, Dako, Glostrup, Denmark), followed by EnVision reagent anti-rabbit IgG horseradish peroxidase-conjugated polymers. Each incubation step was followed by washes in TBS containing 0.5% (*v/v*) Triton X-100. Finally, the reactions were visualized by incubating the sections with NovaRED (Vector Laboratories, Burlingame, CA, USA) according to the manufacturer´s instructions and counterstained with Mayer’s hematoxylin.

The specificity of uPAR immunoreactivity was verified by the following controls: (1) Pre-incubation of the anti-mouse uPAR pAb with a 10-fold molar excess of purified recombinant soluble mouse uPAR for 2 h at room temperature; and (2) omission of the pAb against mouse uPAR (i.e., only the secondary antibody was added to the tissue sections).

### 2.8. In Vitro Transcription and In Situ Hybridization for uPA 

Antisense and sense uPA probes were generated as described [38]. The ^35^S-radioactivity of the probes was adjusted by dilution in 10 mM DTT and deionized formamide to 750,000 cpm/µL. Then, 3-µm paraffin sections were deparaffinized with xylene, hydrated and washed with 130 mM NaCl, 7 mM NaH_2_PO_4_ (pH 7.0), and pre-treated with TEG-buffer for 10 min at 98 °C under RNAse-free conditions, as described [39].

### 2.9. Co-Culture Conditions of Human Gastric Cancer Cell Lines and H. pylori Strains In Vitro

The human gastric adenocarcinoma cell lines AGS (ATCC #CRL-1739) and MKN45G [40] were cultured in RPMI 1640 GlutaMAX (GIBCO, Life Technologies, Carlsbad, CA, USA) without antibiotics, but supplemented with 6% heat-inactivated fetal calf serum (GIBCO) at 37 °C and 5% CO_2_. Confluent cultures were trypsinized, and 5 × 10^6^ cells were added to 10-cm tissue culture dishes and allowed to reconstitute overnight, reaching approximately 80% confluence.

Suspensions of freshly harvested *H. pylori* strains SS1 or J99 were adjusted to an optical density of 600 nm = 0.25 in RPMI 1640 GlutaMAX without fetal calf serum. Then, 10 mL of bacterial suspensions (corresponding to 1.0 × 10^9^ CFU) or the corresponding buffer controls were added to tissue culture dishes containing adherent MKN45G or AGS cells. After 6 h of incubation at 37 °C, the co-cultures were washed with PBS and cells were harvested using a cell scraper and collected three times in 300 µL 0.1 M Tris pH 8.1, 10 µg/mL trasylol, and 1 mM phenylmethylsulfonyl fluoride (PMSF). The cell pellets were collected by centrifugation at 12.000× *g* for 30 min at 4 °C and immediately lysed by re-suspension in 900 µL of lysis buffer (0.1 M Tris pH 8.1, 0.5% (*w/v*) deoxycholat, 1% (*v/v*) NP40, 0.1% (*w/v*) SDS, 10 mM EDTA, 1 mM PMSF, and 10 µg/mL trasylol). Lysis proceeded on ice for 60 min with short pulses of vortexing every 20 min. Supernatants were isolated by centrifugation at 15.000× *g* for 20 min at 4 °C.

Detection of uPAR expression in these detergent lysates was assessed by SDS-PAGE of 10 µL of lysate (~6 × 10^4^ cells) followed by electroblotting onto PVDF membranes. Excess protein binding sites were blocked by incubation with 2% (*w/v*) skim milk powder. Visualization of uPAR expression was subsequently accomplished by first an overnight incubation at 4 °C with 0.1 µg/mL of the primary rabbit anti-uPAR pAb and a second 1-h incubation at room temperature with a 5000-fold dilution of secondary HRP-conjugated swine anti rabbit-Ig pAb (Code P 0217, DAKO, Glostrup, Denmark). Specificity was validated by pre-incubation of the primary rabbit anti-uPAR antibody with a 10-fold molar excess of purified soluble uPAR expressed in *Drosophila* S2 cells [41], before addition to the PVDF membrane. Equal loading conditions were assessed after stripping the membrane and reprobing the blots with a mouse anti β-actin mAb (code: mAbcam 8226; Abcam, Cambridge, UK).

### 2.10. H. pylori Colonization, Histopathology and Immunohistochemistry Evaluations

The *H. pylori* colonization was evaluated according to the number of gastric glands containing bacteria and the density of bacteria, as proposed [42]. Histopathological evaluations of the mouse gastric mucosa and uPAR expression were scored by two independent observers and graded using published guidelines [43], focusing particularly on inflammation and metaplasia. Scoring was done in a blinded manner by the two observers and a consensus was subsequently reached before the identity of the samples were decoded. Infiltration of inflammatory cells in the gastric mucosa was determined by counting the absolute number of CD3-positive and F4/80-positive cells per microscopic visual field (approximately 0.2 mm^2^) using the ImageJ software (available at: https://imagej.nih.gov/ij/). A cell proliferation index was estimated as the proportion of Ki67-positively stained nuclei per gastric gland in at least 5 well-oriented glands. Semi-quantitative assessment of uPAR expression was based on the intensity of staining in gastric epithelial cells according to the following categories: 0, negative; 1, weak; 2, moderate; 3, intense; and 4, very intense. Scorings for all parameters was performed independently at five anatomical locations of the mouse stomach: The squamo-columnar junction, two sites of the corpus (proximal and distal segments), the region encompassing the transitional mucosa between the corpus and antrum (denoted mid-stomach), and the antrum. Since statistical analysis revealed very similar results for the two anatomical sites of the corpus, we refer to the corpus as a single entity and all our statistical analyses correspond to data collected for the proximal corpus only.

### 2.11. Statistical Analysis

The association between variables on an ordinal scale is presented by the Spearman rank correlation. The general linear model was used for the analysis of continuous variables (CD3, F4/80, and Ki67) with time PI, antimicrobial treatment, or PGE_2_ treatment as explanatory variables. For categorical variables on an ordinal scale (inflammation and uPAR scores), chi-square tests for trend were used to compare pre-specified pairwise comparisons with exact *p*-values. The analysis of categorical variables on an ordinal scale and time of measurement were done using a proportional odds regression analysis. *p*-values less than 5% were considered significant. All calculations were done using SAS (v. 9.2, SAS Institute, Cary, NC, USA).

## 3. Results

### 3.1. Histopathological Changes Induced by H. pylori Infection

**Figure 2:**
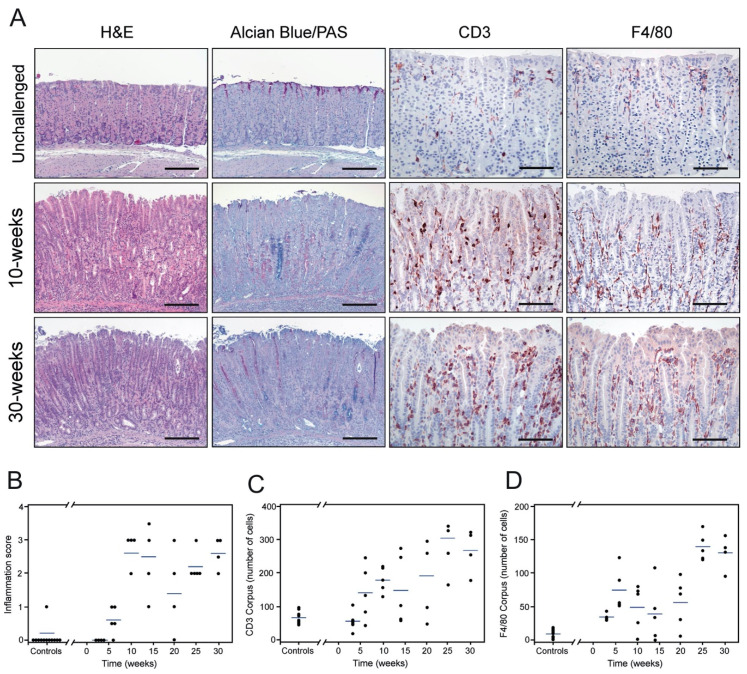
Histopathology of the murine gastric corpus mucosa with chronic *H. pylori* infection. Adjacent tissue sections from resected gastric mucosa of unchallenged and *H. pylori*-inoculated mice were stained by H&E and Alcian Blue/PAS or processed for immunohistochemical detection of CD3 and F4/80 (**A**). Unchallenged mice (upper row) show a normal mucosal architecture. Ten weeks after *H. pylori* inoculation (middle row), pronounced histological alterations are observed in the corpus, including inflammation (CD3 and F4/80) and mucous metaplasia with shifts towards intestinal-type acidic mucins (blue) and gastric-type neutral mucins (pink) in the oxyntic mucosa (Alcian Blue/PAS staining). This pathology is further exacerbated on sustained infection at 30 weeks (lower row). Histopathological assessment of inflammation (scored according to the scheme proposed by [43]) (**B**), infiltrating CD3-positive (**C**) and F4/80-positive cells (**D**) in the mucosa of non-infected (controls, *n* = 9) and *H. pylori*-challenged mice at different time-points (one experiment; *n* = 4–5 mice per timepoint) with the blue line representing mean values. Scale bars in A: H&E and Alcian Blue/PAS microphotographs ≈ 200 µm, CD3 and F4/80 microphotographs ≈ 100 µm.

*H. pylori* infection causes a number of well-described histopathological changes in the gastric epithelium of colonized mice [43]. We analyzed the occurrence of these lesions in cohorts of *H. pylori*-infected mice from 3 to 30 weeks PI. First, we interrogated the *H. pylori* colonization status of the animals by immunohistochemistry and found bacteria in the luminal space along the gastric epithelium at all time-points. Bacterial clusters of variable density were generally observed in close proximity to or in direct contact with epithelial cells in the upper third of the mucosa, in particular at the proximal segment of the corpus (Supplementary Figure S1). Three weeks after colonization, *H. pylori*-infected mice had normal gastric mucosal architecture with very few scattered inflammatory cells within the lamina propia, comparable to non-infected animals (Supplementary Figure S2). Six weeks after challenge, a minor influx of inflammatory cells was observed focally at the base of the gastric glands (Figure 2C,D), while in general, the mucosal architecture resembled that of unchallenged mice (Figure 2B). Distinct histopathological lesions were first observed in *H. pylori*-infected mice after 10 weeks of colonization, particularly in the corpus. At this time-point, overt multifocal infiltration of inflammatory cells in the mucosa and submucosa was observed (Figure 2A). CD3-positive cells (T-cells) were identified as the main inflammatory cell type recruited to these compartments of the gastric wall (Figure 2A). Other infiltrating immune cells were also seen, including F4/80-expressing cells (primarily macrophages) and neutrophils (Figure 2A). In addition, these mice exhibited functional loss of parietal cells, which were replaced by mucous-secreting cells, accompanied by alterations in the production of gastric mucins (Figure 2A), all being morphological hallmarks of a developing mucous metaplasia. Inflammation and mucous metaplasia were further exacerbated at 25 weeks PI (Figure 2B–D). Thirty-week-infected mice displayed robust infiltration of inflammatory cells and manifest mucous metaplastic lesions encompassing large areas of the corpus and mid-stomach (Figure 2A). Foci of pseudopyloric metaplasia were observed in the most proximal part of the corpus (not shown). In contrast, no overt histopathological alterations were observed in the antral region of the mouse stomach at any time-point (not shown). Histopathological assessment revealed an overall continuous progression of the inflammation during the course of infection (Figure 2B, *p* = 0.002). Concordantly, the number of infiltrating CD3-positive and F4/80-positive cells in the corpus correlated to the duration of infection (Figure 2C,D, *p* < 0.0001; r_s_ = 0.72, and *p* < 0.0001; r_s_ = 0.70, respectively). A similar correlation was established for the mid-stomach (*p* < 0.0001; r_s_ = 0.60 for CD3 and *p* = 0.0002; r_s_ = 0.55 for F4/80).

### 3.2. H. pylori Infection Induces uPAR Expression in Gastric Epithelial Cells

**Figure 3:**
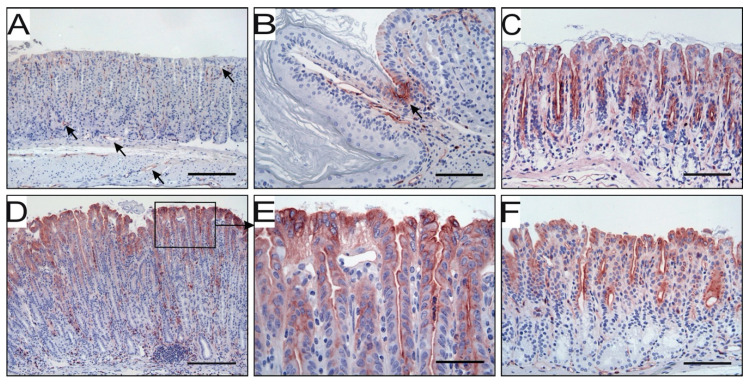
uPAR expression at different anatomical locations in non-infected and *H. pylori*-challenged mice. In the corpus of unchallenged mice, uPAR is detected by immunohistochemistry in endothelial cells (arrows) and a few scattered neutrophils (**A**), as well as in some transitional cells of the squamo-columnar junction (**B**). Epithelial cells in the corpus are negative for uPAR (**A**), while some expression is found in antral epithelium (**C**). Thirty weeks after *H. pylori* inoculation, intense uPAR expression appears in the corpus epithelium (**D**) at the apical membrane of foveolar epithelial cells (**E**). Epithelial cells of the antrum remain uPAR positive in *H. pylori*-infected mice (**F**). Scale bars: A and D ≈ 200 µm; B, C and f ≈ 100 µm; E ≈ 50 µm.

We have previously shown that uPAR expression is upregulated in foveolar epithelial cells of human gastric mucosa colonized with *H. pylori* [29]. To further explore this finding, we tested whether it is recapitulated in experimental mouse models of *H. pylori*-induced gastritis. In unchallenged animals, uPAR is only confined to endothelial cells and a few scattered neutrophils within the lamina propia of the mucosa and submucosal layer of the stomach wall (Figure 3A). These two cell types constitutively express uPAR under normal homeostatic conditions [10]. Intriguingly, we always observed a pronounced and persistent uPAR staining in a small cluster of epithelial cells delimiting the junction between squamous and columnar stomach in unchallenged mice (Figure 3B), but no uPAR expression occurred in the epithelial compartment of the corpus and mid-stomach in non-infected animals (Figure 3A). Epithelial cells located in the antral region were nevertheless positive for uPAR in unchallenged animals (Figure 3C). In mice infected with *H. pylori* for 30 weeks, uPAR expression persisted in endothelial cells and infiltrating neutrophils, whereas uPAR expression in transitional cells residing in the squamo-columnar junction surprisingly disappeared (not shown). More importantly, intense de novo uPAR immunoreactivity appeared in the columnar epithelium of the corpus and mid-stomach in *H. pylori*-colonized mice (Figure 3D). This uPAR expression was confined to the apical surface of foveolar epithelial cells located in the upper third of the gastric units (pits) (Figure 3D,E). Other cell types of the epithelial layer, such as mucous neck, parietal, and chief cells, located in the neck and base of the gastric glands remained uPAR negative (Figure 3D). In contrast, the baseline expression of uPAR in epithelial cells of the antrum remained insensitive to the *H. pylori* infection (Figure 3F).

More distal regions of the gastrointestinal tract were also examined in our mouse model. In both uninfected and *H. pylori*-challenged animals, uPAR was present in endothelial and infiltrating inflammatory cells, presumably neutrophils, within the lamina propia of the crypts, but never in the epithelial compartment of the small intestine and colon (Supplementary Figure S3A–C).

The specificity of our immunohistochemical detection of mouse uPAR was validated by pre-absorption controls (Supplementary Figure S3D–I).

### 3.3. uPAR Expression Levels, Inflammation, and Cell Proliferation Increase with the Duration of H. pylori Infection

**Figure 4:**
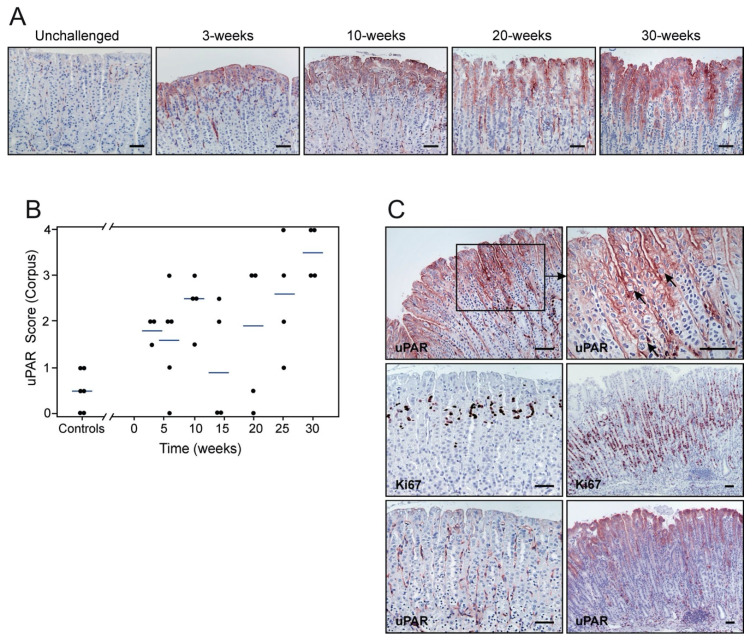
Kinetics of *H. pylori*-induced uPAR expression and cell proliferation in the gastric epithelium. Sections from unchallenged, 3-, 10-, 20-, and 30-week post-inoculation (PI) mice were examined by immunohistochemistry for uPAR (**A**,**C**) and Ki67 (**C**) expression. Semi-quantitative assessments reveal a progressive increase in uPAR intensity (**B**; *p* = 0.006; r_s_ = 0.57) (one experiment; *n* = 9 controls; *n* = 4–5 *H. pylori*-challenged mice per timepoint), which is correlated with cell proliferation (*p* < 0.0001; r_s_ = 0.70). Corresponding uPAR and Ki67 staining on adjacent sections from non-infected (left) and infected (right) mice are shown (**C**). Arrows indicate the presence of mitotic figures. Scale bars ≈ 100 µm.

The kinetics of uPAR induction by *H. pylori* was delineated by a semi-quantitative scoring of uPAR immunostaining at different anatomical locations of the mouse stomach. This revealed a progressive overall increase in the intensity of uPAR staining in foveolar epithelial cells of the corpus as a function of the duration of infection (Figure 4A,B, *p* = 0.006; r_s_ = 0.57). In the antral region, in contrast, the correlation between these two parameters was weak but negative (Supplementary Figure S4B, *p* = 0.03; r_s_ = −0.22). At the squamo-columnar junction, an unexpected negative correlation existed between uPAR expression and the time post-infection (Supplementary Figure S4A, *p* < 0.0001; r_s_ = −0.70). Given the paired increment of both inflammation and uPAR expression, the potential link between these two parameters was further scrutinized. A strong correlation between the number of infiltrating CD3-positive cells and the intensity of uPAR expression in epithelial cells was revealed for the corpus and mid-stomach (*p* < 0.0001, r_s_ = 0.65 for corpus; Figure 2C vs. Figure 4B). Similarly, the number of infiltrating F4/80-positive cells and uPAR expression in epithelial cells at these anatomical sites were highly correlated (*p* < 0.0001, r_s_ = 0.74 for corpus; Figure 2D vs. Figure 4B).

The uPAR staining in foveolar epithelial cells of *H. pylori*-colonized mice was particularly intense in areas of the gastric mucosa where mitotic figures were abundant (Figure 4C). Accordingly, we estimated a cell proliferation index, based on Ki67 staining, and explored if a correlation between uPAR expression and epithelial cell proliferation existed. Cell proliferative activity in the corpus and mid-stomach was substantially enhanced in *H. pylori*-colonized mice, compared to unchallenged mice (Figure 4C), and increased according to time PI (*p* = 0.0003; r_s_ = 0.54 for corpus). Notably, a strong correlation existed between cell proliferation and uPAR expression in the corpus mucosa (*p* < 0.0001; r_s_ = 0.70), and in the mid-stomach (*p* = 0.0002; r_s_ = 0.62). It should, nonetheless, be emphasized that uPAR expression and cell proliferation take place at almost non-overlapping anatomical locations of the epithelial lining (Figure 4C).

### 3.4. uPA is Not UpRegulated in Gastric Mucosa Upon H. pylori Infection

The observed induction of uPAR in the mouse gastric epithelium in response to *H. pylori* infection prompted us to investigate whether a parallel induction of its cognate binding partner, uPA, was evident. In situ hybridization demonstrated uPA mRNA signal in all anatomical locations of the mouse stomach in both non-infected and *H. pylori*-infected mice after 6, 10 and 14 weeks of PI (Supplementary Figure S5A,D,G and Supplementary Figure S5B,E,H, respectively). Negative controls using the corresponding sense probes showed no uPA signal (Supplementary Figure S5C,F,I). In general, uPA-positive cells were located within the lamina propia of the mucosa, especially at the luminal edge of the gastric units, whereas epithelial cells were devoid of uPA mRNA signal (Supplementary Figure S5D–E, Supplementary Figure S5G–H).

### 3.5. Antimicrobial Eradication of H. pylori Infection Downregulates uPAR Expression

**Figure 5:**
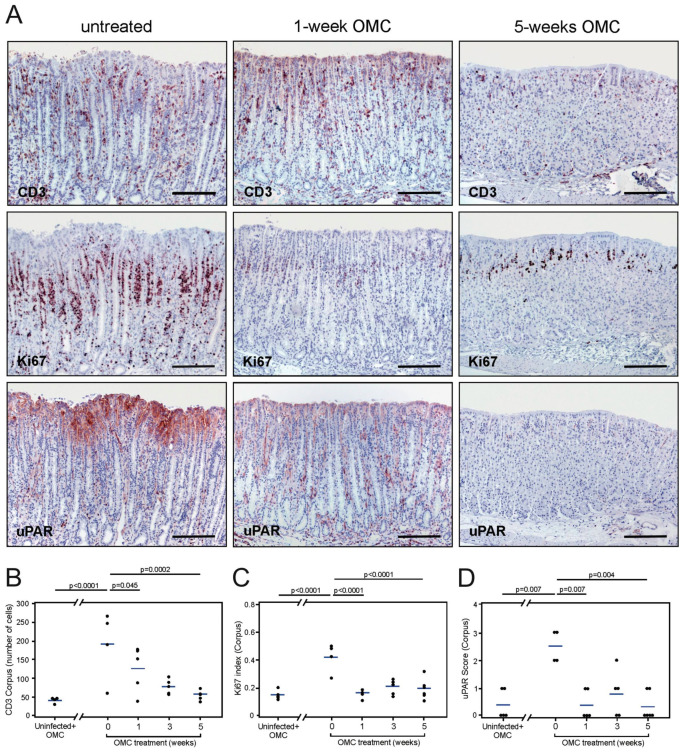
Effect of antimicrobial therapy on uPAR expression in the mouse gastric epithelium. Adjacent sections from 14-week *H. pylori*-infected mice untreated (**A**, left column) or treated with antibiotics (**A**, middle and right column) were processed for CD3, Ki67, and uPAR expression. Semi-quantitative assessments of infiltrating CD3-positive cells (**B**), cell proliferation activity (**C**), and uPAR staining intensity (**D**) in the corpus of the mouse stomach for each of the studied groups. One experiment with *n* = 4–5 mice per experimental group. Scale bars: CD3, Ki67, and uPAR microphotographs in A ≈ 200 µm. OMC; omeprazole, metronidazole, clarithromycin.

To clarify the mechanism responsible for induction of uPAR expression in response to *H. pylori* infection, we investigated whether a sustained colonization was required to drive the expression of uPAR in the gastric epithelium. In this line of experiments, mice were inoculated with *H. pylori* (one single dose) and a manifest infection was allowed to establish for 14 weeks before initiating the antimicrobial therapy against *H. pylori*. Animals were sacrificed 1, 3, and 5 weeks after treatment and compared to *H. pylori*-colonized sham-treated animals and uninfected mice. *H. pylori* bacteria were observed lining the gastric epithelium of all inoculated and sham-treated animals in the experimental cohort, except for those receiving the antibiotic treatment (not shown). Histological evaluation of infected and sham-treated animals showed significant inflammation of the stomach (Figure 5A,B and Supplementary Figure S6), increased cell proliferation (Figure 5A,C), and developing mucous metaplasia (Supplementary Figure S6). These histopathological aberrations of the gastric mucosa persisted one week after termination of the anti-*H. pylori* therapy, and normal mucosal architecture was gradually restored, reaching full restoration after 5 weeks (Figure 5A–C and Supplementary Figure S6).

Concordant with our above-described experiments, intense uPAR expression was induced in foveolar epithelial cells of the corpus and mid-stomach in *H. pylori*-colonized and sham-treated mice (Figure 5A,D). Importantly, uPAR staining in foveolar epithelial cells at these two anatomical regions was indeed very weak, if present at all, in mice undergoing antimicrobial therapy for *H. pylori* eradication (Figure 5A,D). The expression of uPAR in the antral epithelium remained at the baseline level in inoculated mice irrespective of the treatment (not shown). These results demonstrate that persistent *H. pylori* colonization of the mouse gastric epithelium is required for sustained induction of uPAR expression in foveolar epithelial cells of the corpus and mid-stomach. Intriguingly, uPAR expression in transitional cells residing in the squamo-columnar junction reappeared in parallel with successful *H. pylori* eradication (not shown).

### 3.6. Treatment with PGE_2_ Attenuates Gastric Immunopathology but Does Not Abrogate uPAR Expression in H. pylori-infected Mice

Treatment of *H. felis*-infected mice with synthetic PGE_2_ analogs strongly attenuates inflammation without perturbing the chronic bacterial colonization [36]. We then reasoned we could use PGE_2_ synthetic analogs in our mouse model as a means to uncouple the intrinsic connection between *H. pylori* infection and inflammation. More specifically, we considered this approach well-suited to interrogate whether the observed induction of uPAR is caused by the direct interaction of *H. pylori* with the gastric epithelial cells, or is a bystander phenomenon elicited by the inflammatory response mounted against the infection.

**Figure 6:**
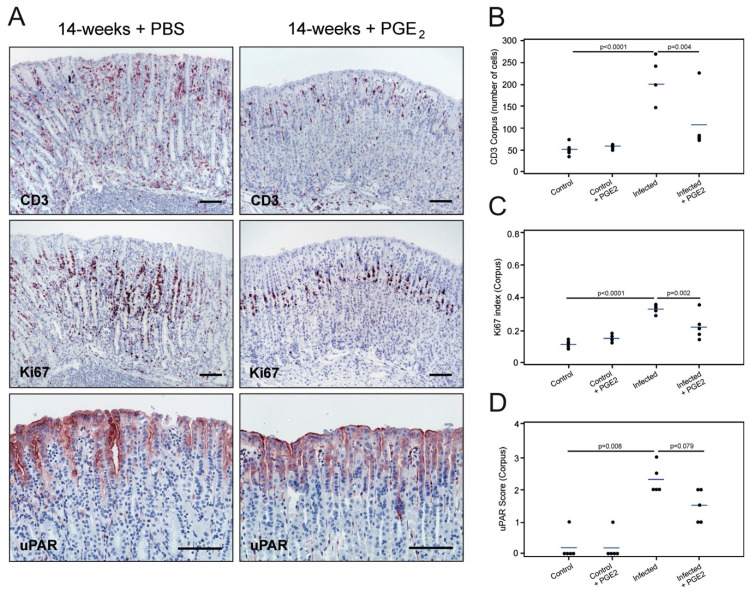
Effect of PGE_2_ treatment on uPAR expression in gastric epithelium colonized with *H. pylori.* Adjacent sections from the gastric mucosa of 14-week *H. pylori*-inoculated mice treated with saline (PBS) (**A**, left column) or with PGE_2_ analogs (**A**, right column) were processed for immunohistochemistry (CD3, Ki67, and uPAR). Semi-quantitative assessment of infiltrating CD3-positive cells (**B**), cell proliferation activity (**C**), and uPAR staining intensity (**D**) in the corpus region of the mouse stomach for each of the studied groups. One experiment with n = 4–5 mice per experimental group. Scale bars 100 µm.

In agreement with the findings reported in [36], we found increased *H. pylori* colonization in the stomachs of PGE_2_-treated mice compared to sham-treated animals (semi-quantitative average score for bacterial colonization: 2.4 for PGE_2_-treated and 1.4 for untreated mice). Untreated *H. pylori*-infected mice exhibited the usual histopathology 14 weeks after challenge, including robust infiltration of inflammatory cells in the mucosa and submucosa, mucous metaplastic lesions, and enhanced epithelial cell proliferation in the corpus and mid-stomach (Figure 6A–C and Supplementary Figure S7). These animals also showed a pronounced uPAR staining in the foveolar epithelial cells of the corpus (Figure 6A,D). In contrast, *H. pylori*-infected mice undergoing PGE_2_ treatment showed almost normal mucosal architecture, substantial downregulation of the inflammatory response, unaltered mucin production, and a cell proliferation activity comparable to that of non-infected animals (Figure 6A–C and Supplementary Figure S7).

This remission was accompanied by a reduction in the expression of uPAR in epithelial cells of the corpus mucosa after treatment with PGE_2_ analogs, although it did not reach statistical significance (Figure 6A,D; *p* = 0.079). Although these data suggest that *H. pylori*-driven inflammation does contribute to the induction of uPAR expression in mouse gastric epithelium, a remnant uPAR protein expression did persist after treatment, which may indicate that the direct interaction of *H. pylori* with the epithelial lining could represent an additional contributing factor to the induction of uPAR protein expression.

### 3.7. H. pylori Induces uPAR Expression in Co-Cultured Gastric Adenocarcinoma Cell Lines

**Figure 7:**
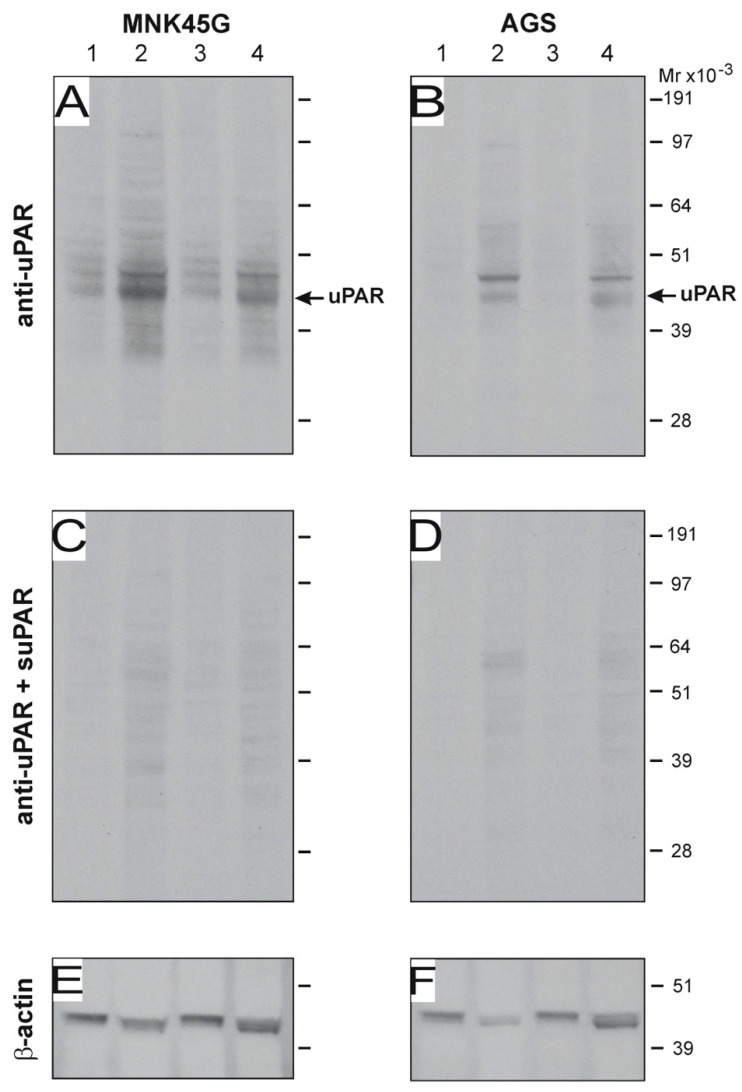
*H. pylori*-induced uPAR expression in gastric cancer cell lines in vitro. The gastric carcinoma cell-lines MNK45G and AGS (5 × 10^6^ cells) were co-cultured with 1 × 10^9^ CFU of *H. pylori* strains SS1 and J99 in serum-free RPMI media for 6 h at 37 °C. Western blotting of cells lysates and corresponding sham controls were developed with an anti-uPAR pAb (**A**,**B**), and the specificity of this detection is verified by pre-absorption with purified uPAR (**C**,**D**). After stripping, the equal loading was probed by staining for β-actin (**E**,**F**). Sham-treated control cells (lanes 1 and 3); cells co-cultured with SS1 (lane 2); cells co-cultured with J99 (lane 4). Su-PAR; soluble uPAR. Experiment performed in triplicate.

Several in vitro studies indicate that *H. pylori* induces uPAR expression in gastric adenocarcinoma cell lines [44,45]. In conjunction with the fact that PGE_2_ analogs do not eliminate uPAR expression in vivo, this prompted us to interrogate the induction of uPAR protein in vitro by exposure to the mouse-adapted SS1 strain of *H. pylori*. We co-cultured a variety of *H. pylori* strains, including SS1, with the gastric adenocarcinoma cell-lines AGS and MNK45G and analyzed the uPAR content in the corresponding total cell lysates. This minimalistic setup shows that, at least in vitro, *H pylori* strain SS1 did indeed induce uPAR expression in both cell lines after only 6 h of co-cultivation (Figure 7). In line with previous studies [46], *H. pylori* strain J99 also induced uPAR expression in our in vitro setup.

## 4. Discussion

*H. pylori* infection is the most well-established risk factor for several gastric pathologies, such as gastritis, peptic ulcer disease, MALT lymphoma, and gastric cancer [3]. Nevertheless, many aspects of the pathogenesis of this bacterial infection still remain enigmatic. Only a small proportion of *H. pylori*-infected individuals develop the above-mentioned pathologies. This feature defines one of the most intriguing paradoxes about this bacterial infection. Presumably, the interaction between *H. pylori* virulence factors, the host response, and the environment dictates the final clinical outcome. Circumstantial evidence suggests that this bacterial infection may be involved in the induction of components of the plasminogen activation system in the gastric mucosa, in particular uPAR [27,29]. This phenomenon may be relevant for the development of gastric pathologies, given the central role of uPAR in plasminogen-mediated extracellular matrix remodeling [47,48,49], vitronectin-dependent cell adhesion and migration [14,15], and its upregulation in gastric cancer and associated prognostic impact [11,29,49].

In the present study, we used a mouse model of *H. pylori*-induced gastritis to explore the relationship between *H. pylori* infection and uPAR expression in vivo, including a spatio-temporal resolution of uPAR induction during *H. pylori* colonization as well as during its eradication by pharmacological intervention. We now demonstrate that *H. pylori* infection plays an important role in the induction of uPAR protein expression in foveolar epithelial cells of the mouse gastric mucosa, which is mainly a reaction of the inflammatory response triggered by the infection. The anatomical location of this aligns excellently with previous findings in human non-neoplastic gastric mucosa showing that uPAR is highly expressed by surface epithelial cells of *H. pylori*-infected patients, as interrogated locally by immunohistochemistry [29], or globally by measuring uPAR mRNA levels [27]. By studying the kinetics of *H. pylori*-driven uPAR induction, we now reveal four new properties: i) The induction of uPAR expression is confined to the foveolar compartment in the gastric epithelium; ii) it represents an early event in the pathogenesis of the infection; iii) it requires sustained bacterial colonization; and iv) it is associated with the inflammatory-mediated reaction.

We also addressed in our mouse model of *H. pylori*-induced gastritis whether the uPAR’s ligand, uPA, was upregulated in the gastric mucosa, given that some in vitro and in vivo studies claim that uPA is also induced by *H. pylori* [27,44]. We found a baseline expression of uPA mRNA by in situ hybridization in cells located within the lamina propia, which remained insensitive to presence of *H. pylori*, the degree of inflammation, and the duration of infection; no uPA expression was seen in the gastric epithelial cells whatsoever. Thus, it is likely that the induction of uPAR in the gastric epithelium in response to *H. pylori* infection does not lead to activation of plasminogen since the uPA-expressing cells are located in a separate compartment of the gastric mucosa.

One of the strategies *H. pylori* uses to secure survival is to colonize less acidic regions of the stomach [50,51]. In humans and mice, *H. pylori* is usually found in the mucous layer lining the gastric epithelium and/or anchored to the apical membrane of foveolar cells in the upper third of the mucosa [51,52,53]. In the present study, the foci in the epithelial compartment where uPAR is upregulated coincide with such preferred niches of *H. pylori* colonization, thus recapitulating the situation found in humans [29]. Nonetheless, the anatomical regions of the stomach preferred for the early phases of *H. pylori* establishment differ between humans and mice. While colonization usually starts in the human antrum [1], *H. pylori* primarily settle in the most proximal part of the murine columnar stomach, close to the squamo-columnar junction, and expand progressively through the corpus [43]. This route of colonization perfectly mirrors that for the induction of de novo uPAR protein expression, which may imply a causative correlation between the two.

uPAR expression is increased in chronic inflammatory conditions, including wound healing, rheumatoid arthritis, Crohn’s disease, chronic ulcerative colitis, and chronic hepatic inflammation [37,47,54,55,56]. In fact, the severity of collagen-induced arthritis in mice is causally linked to uPAR expression as the severe phenotype in these mice is mitigated upon genetic ablation of uPAR, or pharmacological intervention with monoclonal antibodies [57,58]. In most solid cancers, uPAR is focally upregulated at the invasive front of the malignant tumor, which is generally characterized by a robust infiltration of inflammatory cells that are uPAR positive [13]. In the early non-malignant reactive changes of the gastric epithelium, it is the foveolar epithelial cells, and not infiltrating inflammatory cells, that express uPAR. The correlation between uPAR expression and inflammation may nevertheless indicate the presence of either a single driving biochemical stimulator or two separate events sharing almost overlapping kinetics. Our findings in the *H. pylori*-colonized mice systemically treated with synthetic PGE_2_ analogs tend to favor the former scenario, even though uPAR expression is not fully abrogated. Therefore, we cannot rule out the possibility that to a lesser extent *H. pylori* per se induces uPAR expression in epithelial cells. The latter is supported by two independent observations: i) uPAR expression appeared already three weeks PI, before any manifest inflammatory response mounted against *H. pylori* could be detected; and ii) *H pylori* strains do induce uPAR expression in cell lines (further discussed below).

Importantly, we find that eradication of the manifest *H. pylori* infection suppresses uPAR expression even before normal gastric epithelial architecture is restored, demonstrating that sustained colonization is required for the maintenance of elevated uPAR levels in foveolar epithelial cells. In mouse models of *Helicobacter*-induced gastritis, eradication of the bacterial infection with antimicrobial therapy is completed within 24 h [35], and this leads to a subsequent restoration of the normal mucosal architecture [59]. In our model, downregulation of uPAR expression is accomplished already after one week, whereas regression of inflammation and mucous metaplasia gradually occurs after termination of the antimicrobial treatment. The turnover time of foveolar epithelial cells is as short as 3 to 4 days [60], which enables us to resolve and separate the direct impact of *H. pylori* infection/eradication on uPAR induction from the slower development of general gastric pathology.

Although the induction of uPAR in gastric epithelial cells to a large extent is driven by the inflammatory reaction elicited in response to *H. pylori*, several in vitro studies show that in co-cultures, the bacterium induce uPAR expression in gastric adenocarcinoma cell lines [44,45]. Accordingly, global gene expression analyses rank uPAR among the top upregulated genes in response to this infection [23,24]. A few studies report that the induction of uPAR in gastric cancer cell lines is predominantly linked to CagA-positive strains [23,44]. Nonetheless, we provide both in vivo and in vitro evidence showing that uPAR also can be induced by *H. pylori* SS1 strain, which is regarded as *cagA* positive but has lost the functionality of the *cag* PAI upon its adaptation to mouse gastric physiology [61,62,63]. 

We show here in vivo that one of the consequences of *H. pylori* infection is the induction of uPAR protein expression in gastric epithelial cells very early in the course of infection. Importantly, our experimental evidence highlights the persistence of *H. pylori* bacteria in the proximity of the epithelial lining as a necessary event for the induction of uPAR, which adds a new parameter to the pathogenicity of this bacterial infection. Although some in vitro studies have elaborated further on the mechanisms underlying the induction of uPAR in response to *H. pylori* infection [45,46], additional mechanistic studies in vivo are warranted. Additionally, the functional implications of this phenomenon in the development of gastric pathologies remain to be clarified. Circumstantial evidence from mouse models of pulmonary infections implicates uPAR in the host clearance of the causative bacterial pathogens [30,31,32,33]. *H. pylori* utilizes various host cell surface glycoproteins to anchor to the gastric epithelial cells [64,65]. This opens the intriguing possibility that uPAR could play an accessory role in anchoring *H. pylori* to the epithelial cells in vivo via its interplay with integrin function. Future studies scrutinizing this hypothesis experimentally are nevertheless needed.

Although our study provides correlations implying that there is a functional link between uPAR expression and *H. pylori* infection in vivo, some precaution in the interpretations are required. First, mouse models typically show important intra- and inter-group variations, which is in fact one of the weaknesses inherent to these models. Second, the evidence suggesting a connection between uPAR expression and inflammation is indirect based on correlative observations without direct proof of causality. Notwithstanding this limitation, there is ample evidence in the literature demonstrating a causative effect of uPAR expression on the exacerbation of chronic inflammatory conditions [37,47,54,55,56,57,58].

## Supplementary Materials

The following are available online at https://www.mdpi.com/2076-2607/8/7/1019/s1, **Figure S1**: *H. pylori* colonization of the mouse stomach at selected time-points, **Figure S2**: Histology of the murine gastric corpus mucosa three weeks after *H. pylori* colonization, **Figure S3**: uPAR expression in distal locations of the gastrointestinal tract and verification of the specificity of uPAR immunohistochemistry, **Figure S4**: Kinetics of *H. pylori*-induced uPAR expression in the squamo-columnar junction and antrum of the mouse stomach, **Figure S5**: uPA expression at different anatomical locations of the stomach in unchallenged and *H. pylori*-infected mice, **Figure S6**: Histopathology of the stomach of unchallenged and *H. pylori*-infected mice without or with antimicrobial treatment for *H. pylori* eradication, **Figure S7**: Histopathology of the stomach of unchallenged and *H. pylori*-infected mice without or with PGE_2_ treatment.
