# Supplementary material for: Helicobacter pylori Colonization Drives Urokinase Receptor (uPAR) Expression in Murine Gastric Epithelium During Early Pathogenesis
Source: Microorganisms. 2020 Jul 9;8(7):1019. doi: 10.3390/microorganisms8071019 (PMC7409347; doi:10.3390/microorganisms8071019)
Supplement: Supplementary file 1 [file microorganisms-08-01019-s001.pdf]

Supplementary Material

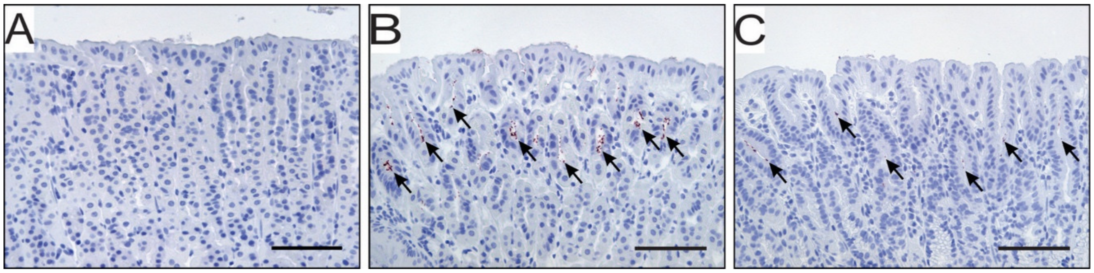

Supplementary Figure 1. *H. pylori* colonization of the mouse stomach at selected time-points. Stomach tissue sections of uninfected **(A)**, 3 weeks PI **(B)**, and 30 weeks PI **(C)** mice were processed for immunohistochemistry against *H. pylori*. No bacteria were observed in unchallenged animals **(A)**. Dense clusters of *H. pylori* bacteria (arrows) were observed in the upper third of the gastric glands along the gastric epithelium of the mouse stomach 3 weeks PI **(B)**. Although less dense, *H. pylori* bacteria were seen at a similar anatomical location in mice 30 weeks post-challenge **(C)**. Scale bars: **A-C** ≈ 100 μm.

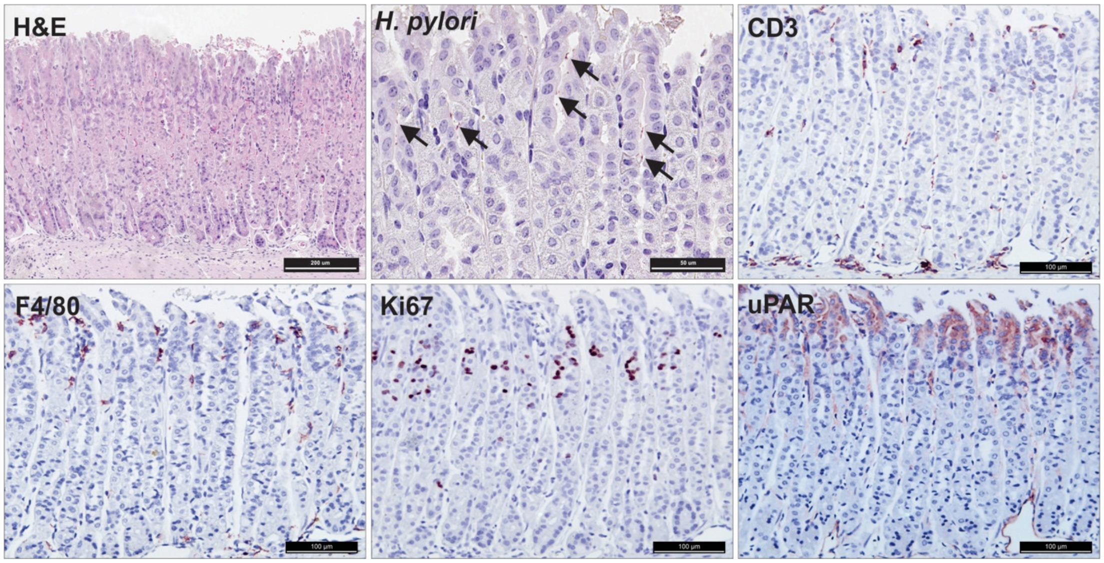

Supplementary Figure 2. Histology of the murine gastric corpus mucosa three weeks after *H. pylori* colonization. Adjacent tissue sections from resected gastric mucosa of 3 weeks PI mice were stained by H&E or processed for immunohistochemical detection of *H. pylori*, CD3, F4/80, Ki67 and uPAR. Despite being colonized by *H. pylori* (arrows), normal mucosal architecture and cell proliferation rate are observed. Few scattered inflammatory cells are seen infiltrating the mucosa. uPAR expression is slightly upregulated in corpus foveolar epithelial cells of 3-week PI mice. Scale bars: H&E ≈ 200 µm; CD3, F4/80, Ki67, and uPAR ≈ 100 µm; *H. pylori* ≈ 50 µm.

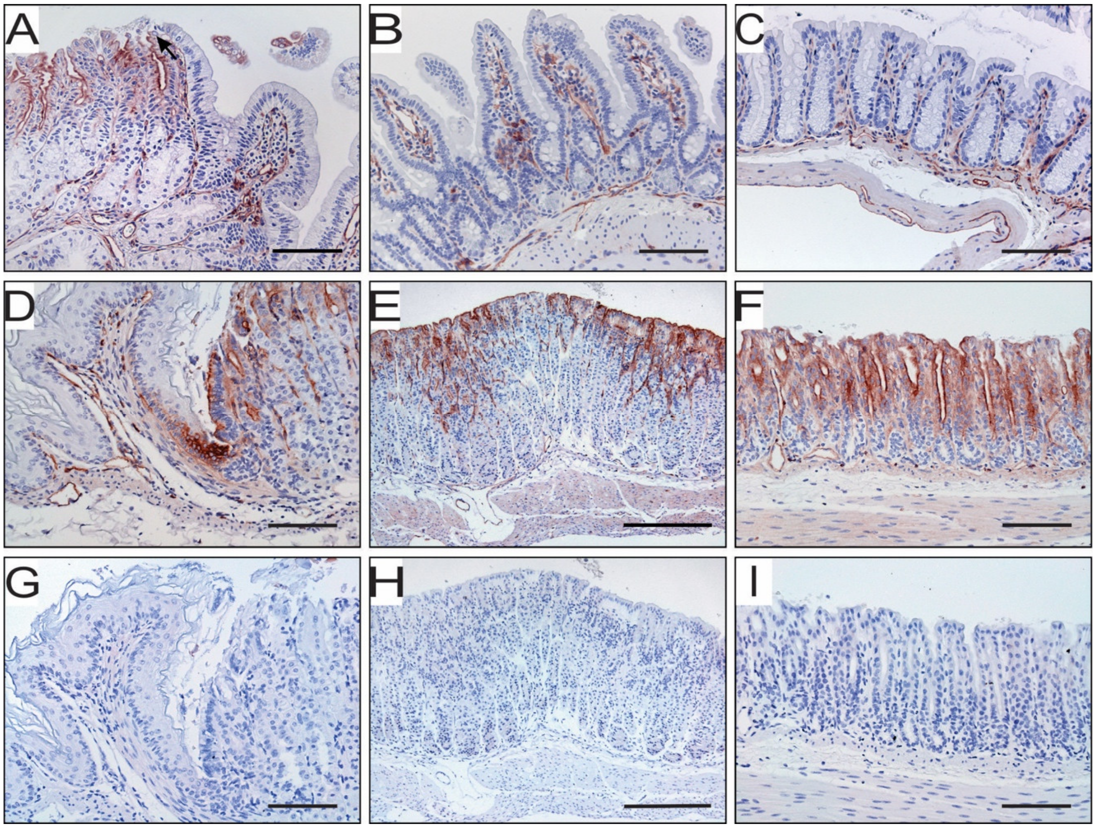

Supplementary Figure 3. uPAR expression in distal locations of the gastrointestinal tract and verification of the specificity of uPAR immunohistochemistry. Tissue sections of the stomach (A,D–I), small intestine (B), and colon (C) of 14 weeks *H. pylori*-infected mice were processed for immunohistochemistry against uPAR. Panels D and G, E and H, and F and I represent neighboring sections. uPAR staining is abruptly halted at the transitional epithelium of the antro-pyloric junction (A, arrow). uPAR staining is observed in endothelial and inflammatory cells, presumably neutrophils, within the lamina propia but not in the epithelial layer of the small intestine (B) and colon (C). Intense uPAR immunoreactivity was observed in the squamo-columnar junction (D), corpus (E), and antrum (F) of *H. pylori*-colonized mice. uPAR staining is precluded at similar anatomical locations by pre-incubating the anti-uPAR pAb with 10-fold molar excess of recombinant mouse uPAR, prior to addition to the tissue sections (G–I). Scale bars: A–D, F, G, I ≈ 100 µm; E and H ≈ 200 µm.

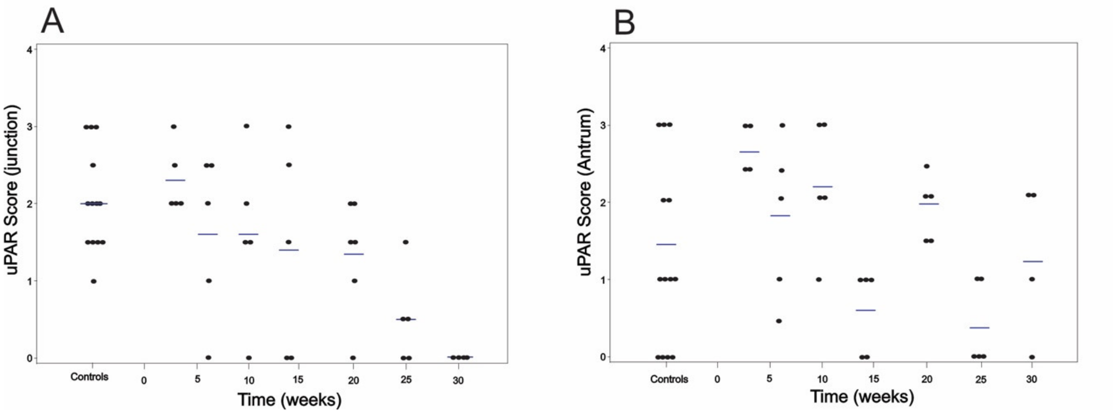

Supplementary Figure 4. Kinetics of *H. pylori*-induced uPAR expression in the squamo-columnar junction and antrum of the mouse stomach. Semi-quantitative assessments reveal a progressive decrease in uPAR intensity in the squamo-columnar junction (A; p<0.0001; r_s= -0.70). In the antrum, uPAR intensity tends to decrease as a function of the duration of infection (B; p= 0.03; r_s = -0.22). One experiment; n=13 controls; n= 4-5 *H. pylori*-challenged mice per timepoint)

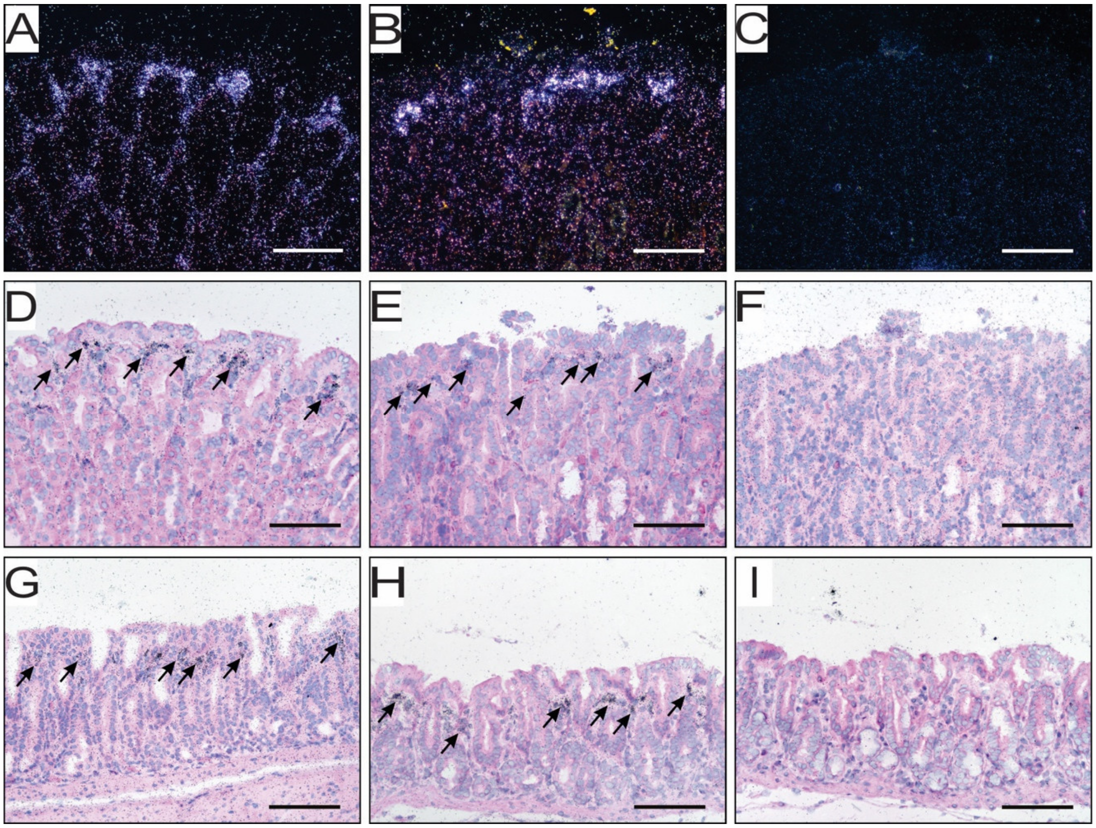

Supplemental Figure 5. uPA expression at different anatomical locations of the stomach in unchallenged and *H. pylori*-infected mice. Stomach tissue sections from unchallenged (A,D,G) and 14 weeks *H. pylori*-colonized (B–C,E–F,H–I) mice were processed for in situ hybridization with antisense (A–B,D–E,G–H) or sense (C,F,I) probes against uPA mRNA. B and C, E and F, and H and I represent adjacent sections. uPA mRNA signal (arrows) was observed in cells located within the lamina propia of the gastric mucosa, especially in the upper third of the gastric units in the corpus of both uninfected (A and D) and challenged (B and E) mice. A similar expression pattern was observed in the antral region of unchallenged (G) and *H. pylori*-colonized (H) mice. No specific uPA signal was detected in the corpus (C and F) or antrum (I) when tissue sections were incubated with sense probes. Scale bar: ≈ 100 μm for all panels.

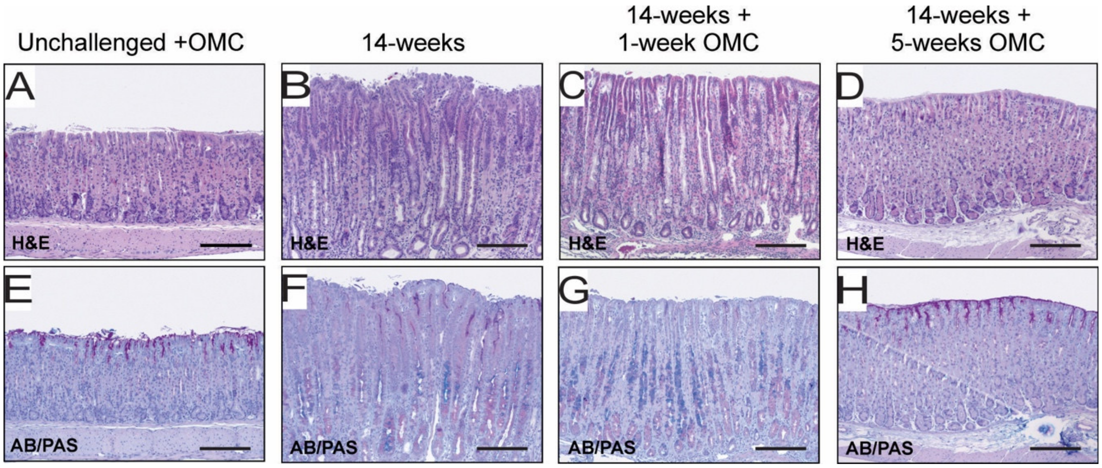

Supplementary Figure 6. Histopathology of the stomach of unchallenged and *H. pylori*-infected mice without or with antimicrobial treatment for *H. pylori* eradication. Stomach tissue sections from unchallenged (left column) and *H. pylori*-infected mice (mid-left, mid-right, and right columns) that were sham-treated (mid-left column) or received antimicrobial treatment for *H. pylori* eradication (mid-right and right columns) were processed for H&E (first row) and AB/PAS (second row) staining. Each column represents adjacent sections. Normal microscopic architecture of the stomach, with no inflammation and normal mucin production, was observed in non-infected mice treated with antibiotics, as revealed by H&E and AB/PAS staining (left column). Important histopathological alterations were seen in *H. pylori*-infected and sham-treated mice after 14 weeks of challenge, including inflammation and mucous metaplasia (mid-left column). These lesions persisted in mice sacrificed one week after termination of the anti-*H. pylori* therapy (mid-right column). Five weeks after eradication treatment, *H. pylori*-infected mice showed no signs of gastric pathology, normal mucosal architecture, no inflammation, and unaltered mucin production (right column). Scale bar: ≈ 200 µm for all microphotographs, OMC; omeprazole, metronidazole, clarithromycin.

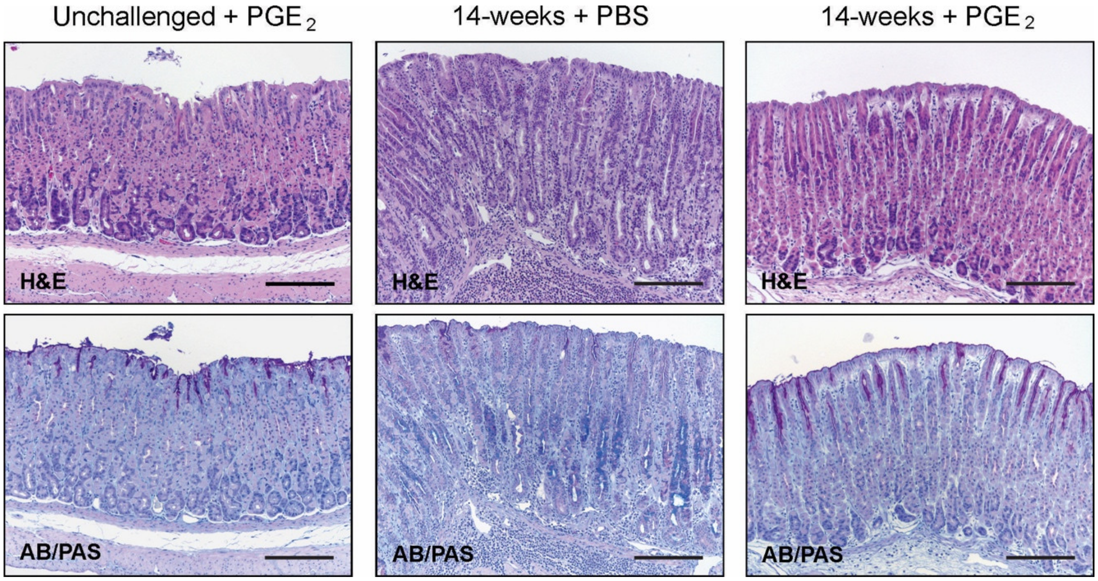

Supplementary Figure 7. Histopathology of the stomach of unchallenged and *H. pylori*-infected mice without or with PGE_2 treatment. Stomach tissue sections from unchallenged (left column) and *H. pylori*-infected (middle and right columns) mice treated with PBS alone (middle column) or PGE_2 analogs (left and right columns) were stained with H&E (first row) or AB/PAS (second row). Each column represents adjacent sections. Normal microscopic mucosal architecture, no inflammation, and a normal mucin expression pattern were observed in non-infected mice treated with PGE_2 (left column). *H. pylori*-infected mice receiving only PBS underwent important histopathological changes after 14 weeks of challenge, including prominent inflammation and altered mucin production (middle column). *H. pylori*-infected mice treated with PGE_2 analogs showed almost normal mucosal architecture, downregulated inflammation, and unaltered mucin production (right column). Scale bar: ≈ 200 μm for all microphotographs.
